# Intragenic deletion of *RBFOX1* associated with neurodevelopmental/neuropsychiatric disorders and possibly other clinical presentations

**DOI:** 10.1186/1755-8166-6-26

**Published:** 2013-07-03

**Authors:** Wei-Wei Zhao

**Affiliations:** 1 Department of Molecular Pathology, KingMed Genome Diagnostic Laboratory, 2429 XinGangDong Road, Haizhu Science and Technology Building, Guangzhou 510330, China

**Keywords:** Microdeletion, Chromosome 16p13.3, *RBFOX1*, Chromosomal microarray analysis (CMA), Seizures, Developmental delay

## Abstract

**Background:**

RBFOX1 is an important splicing factor regulating developmental and tissue-specific alternative splicing in heart, muscle, and neuronal tissues. Constitutional genetic defects in *RBFOX1* are implicated in multiple medical conditions.

**Results:**

We identified 14 copy number variants (CNV) involving *RBFOX1* from 2,124 consecutive pediatric patients referred for chromosomal microarray analysis (CMA), including 13 intragenic deletions and a single intragenic duplication. The clinical significances of the intragenic deletions of *RBFOX1* were evaluated.

**Conclusions:**

Our data strongly supports the associations of intragenic deletions of *RBFOX1* with a diversity of neurodevelopmental and neuropsychiatric disorders, and possibly other clinical features.

## Background

*RBFOX1* [RNA binding protein, fox-1 homolog (C. elegans)], located at human chromosome 16p13.3, is one of the three member in Fox gene family encoding splicing factors [1,2]. It is specifically expressed in heart, muscle and neuronal tissues, regulating developmental and tissue-specific alternative splicing by binding with (U)GCAUG sequence in introns flanking alternative exons of its target genes [1,3-6]. RBFOX1 was initially identified as a binding partner of the brain-specific protein ATXN2, causing spinocerebellar ataxia 2 (SCA2) when the CAG repeats in this gene are expanded. Since then, constitutional genetic defects in *RBFOX1* are implicated in multiple medical conditions including mental retardation and epilepsy [7], bipolar schizoaffective disorder [8], attention-deficit hyperactivity disorder (ADHD) [9], autism [10-13], hand osteoarthritis [14], congenital heart defects [15], obesity and diabetes [16,17], Intracranial arachnoid cysts [18], and primary biliary cirrhosis [19].

## Results and discussion

**Figure 1:**
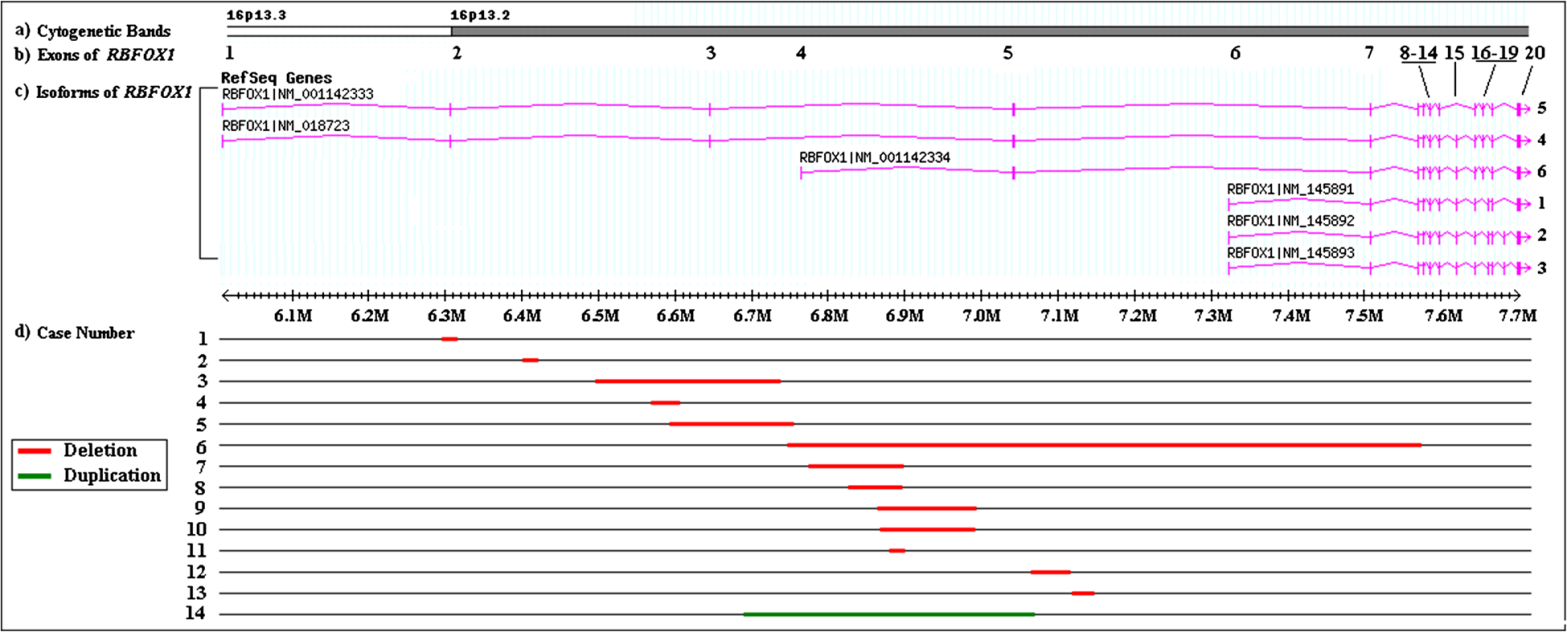
**Genomic overview of the intragenic deletions (patients 1–13) and duplication of *RBFOX1* (patient 14) in this study.**

In this report, we evaluated the clinical significances of the intragenic deletions/duplication of *RBFOX1* in 12 out of 14 patients who carry the genomic imbalances. Fourteen copy number variants (CNV) involving *RBFOX1*, including 13 intragenic deletions and a single intragenic duplication, were identified in this cohort (Figure 1; Table 1), representing ~0.66% (14/2,124) of the patients analyzed in this study. There are no other clinically relevant CNVs in their genomes except in Patient 6 who carries an additional 178 kb deletion of 1p31.3 involving *NF1A* besides the intragenic deletion of *RBFOX*. Of the 13 deletions, 4 of them (in patients 1, 3, 5, and 6) involve exons while the remaining deletions involve only introns. The intragenic duplication in Patient 6 involves exons 4 and 5 of *RBFOX1*. The deletions/duplications were confirmed by qPCR methods (data not shown). The deletion in Patient 5 was maternally inherited while both deletions in Patient 6 occurred de novo. The inheritance patterns of the deletions/duplication could not be determined in remaining patients due to unavailability of DNA samples from either one parent in Patient 8 or both parents in other patients. By comparing breakpoints of the 14 intragenic deletions/duplication within the group and with the CNVs involving *RBFOX1* documented in the DGV (http://projects.tcag.ca/variation/), multiple hot spots involving the generation of CNVs within *RBFOX1* were observed, such as the proximal breakpoints of the CNVs in Patients 7, 8, and 11, and both proximal and distal breakpoints of the CNVs in Patients 9 and 10 (Figure 1; Table 1). In addition, G-banding analysis showed negative results for all 14 patients while a pathogenic mutation in *ZEB2* and a pathogenic mutation in *F8* were identified in Patient 1 and Patient 9 respectively.

**Table 1:** **CNVs involving *RBFOX1*gene on chromosome 16**

**Case**	Start* (bp)	Stop*(bp)	**Size (bp)**	**Del/Dup**	**Affected exon(s)**	**Inheritance and comments**
1	6,296,453	6,317,524	21,071	Del	2	NT, a diagnosis of Mowat-Wilson syndrome
2	6,398,588	6,417,167	18,579	Del	-	NT
3	6,500,668	6,737,047	236,379	Del	3	Inconclusive
4	6,567,938	6,609,613	41,675	Del	-	NT
5	6,597,626	6,759,803	162,177	Del	3	Maternally inherited
6	6,752,063	7,577,445	825,382	Del	4 to 9	An additional 178 kb deletion of 1p31.3 (61,318,736-61,496,706); De novo for both deletions
7	6,781,978	6,948,525	166,547	Del	-	NT
8	6,829,209	6,904,158	74,949	Del	-	NT
9	6,860,713	7,018,893	158,180	Del	-	NT, a diagnosis of mild hemophilia A
10	6,866,819	7,011,349	144,530	Del	-	NT
11	6,883,111	6,912,449	29,338	Del	-	NT
12	7,079,544	7,133,209	53,665	Del	-	NT
13	7,132,809	7,170,657	37,848	Del	-	NT
14	6,689,120	7,062,757	373,637	Dup	4, 5	NT

Notes: *The genomic coordinates of these CNVs were based on the human genome annotation of hg18 build; *Del* deletion, *Dup* duplication, *NT* not tested.

Of the 14 patients containing intragenic deletions/duplication of *RBFOX1* (Tables 1 and 2), two of them were excluded for further analysis of the genotype-phenotype correlations, including Patient 1 who was diagnosed with Mowat-Wilson syndrome both phenotypically and genotypically, and Patient 14, the single case with an intragenic duplication of *RBFOX1* accompanied with multiple congenital anomalies. Although both Patients 6 and 9 contain additional genomic abnormalities in addition to the intragenic deletions of *RBFOX1*, these two cases were included for further analysis since the clinical significance of the 178 kb deletion of 1p31.3 (61,318,736-61,496,706) involving *NFIA* in Patient 6 has not been defined yet and the mild hemophilia A in Patient 9 is assumed not to interfere the phenotypic effects of the *RBFOX1* deletion. Recurrent features in the 12 patients (Patients 2 to 13) with intragenic deletions of *RBFOX1* include global developmental delay (GDD) (7/12), epilepsy (6/12), macrocephaly or microcephaly (6/12), renal problems (4/12), such as single kidney, renal insufficiency, hypospadias, voiding dysfunction and nocturnal enuresis, bone and muscle problems (4/12), such as hemivertebrae, kyphosis, equinovarus, contractures of knees and hips, hypotonia (2/12), and hearing impairment (2/12). Some isolated findings were also observed, including autism, paralyzed vocal cords, tracheomalacia, gastroesophageal reflux, complex congenital heart defect, short stature, type II diabetes, adenoid hypertrophy, hypothyroidism, lactic acidosis, and hypernatremia (Table 2).

**Table 2:** **Clinical findings in patients with intragenic deletions**/**duplication in this study**

**Cases**	**Sex**	**Dysmorphism**	**Neurological problems**	**GDD/Autism/speech delay**	**Other organ abnormalities/disorders**
1	F	None	None	GDD	Hypotonia, feeding problems, eczema, bilateral renal reflux and dilatation, episodes of fussiness and gassiness, chronic constipation, a diagnosis of Mowat-Wilson syndrome
2	M	Macrocephaly	None	GDD, Autism	Hypothyroid at birth, history of lactic acidosis
3	M	None	Epilepsy	None	Gastroesophageal reflux, voiding dysfunction and nocturnal enuresis, and adenoid hypertrophy
4	M	Severe microcephaly	Perinatal hypoxic ischemic encephalopathy with resultant severe cystic encephalomalacia, focal epilepsy, cerebral palsy, asymmetric spasticity	GDD, especially speech delay	None
5	F	None	None	None	Bicuspid aortic valve with aortic dilation, anomalous superior left pulmonary venous return
6	M	Macrocephaly	None	Severe GDD	Hypotonia, kyphosis
7	M	None	Encephalopathy	Alexia, dyslexia	Short stature
8	M	None	Epilepsy	Mild developmental delay, speech delay	Hypospadias
9	M	Macrocephaly	Poorly developed corpus callosum	Severe GDD	Hearing impairment, hypernatremia and renal insufficiency, a diagnosis of mild hemophilia A
10	F	None	None	None	Hypotonia, tracheal malasia,
11	F	Plagiocephaly and infantile torticolli	Epilepsy, abnormal EEG with a slow background and temporal spikes, and staring spells	GDD, especially speech delay	Hearing impairment
12	F	None	Epilepsy	Intellectual disability, aggressive behavior	None
13	M	Severe microcephaly and micrognathia	Epilepsy, ventriculomegaly	None	Paralyzed vocal cords, bilateral equinovarus, contractures of knees and hips bilaterally
14*	M	None	None	NA	Atrioventricular canal, transposition of the great arteries, pulmonary valve atresia, supraventricular tachycardia, and congenital absence of spleen

Notes: *-newborn; *M* male, *F* female, *GDD* global developmental delay, *EEG* electroencephalogram, *NA* not applicable.

We believe the intragenic deletions of *RBFOX1* identified in these patients are risk factors for a wide spectrum of neurodevelopmental disorders and other clinical features. 1. Comparing with healthy groups, the 0.61% (13/2,124) detection rate of intragenic deletions of *RBFOX1* in our study is significantly higher than the rate observed in control populations, such as the 0.1% (2/1854) in Itsara’s report [20] and the 0.2% (4/2026) in Shaikh’s report [21]. 2. Genetic defects including point mutations or intragenic deletions of *RBFOX1* were independently reported to be associated with a variety of clinical conditions [7,9-11,14-19]. Majority of the clinical findings in the 12 patients in our cohort, such as GDD, seizures, and other neurodevelopmental and neuropsychiatric disorders, are consistent with those reported cases who carried genetic defects in *RBFOX1*[7-11,22]. For example, a recent study identified exonic deletions of the *RBFOX1*gene in 5 of the 1,408 European patients with idiopathic generalized epilepsy (IGE), but in none of the 2,256 population controls [22]. Davis LK et al. presented a inherited *RBFOX1* exon-2 deletion in a boy with autism and developmental hemiparesis [23]. Bone and muscle problems present in multiple cases in this study were also observed in a report [14]. 3. The intragenic deletions of *RBFOX1* in patient 6 occurred de novo, providing additional support about its pathological relevance to severe GDD, macrocephaly, hypotonia, and kyphosis in this patient. 4. More importantly, functional studies on *RBFOX1* strongly support the etiological roles of *RBFOX1* for neurodevelopmental disorders [10,24,25]. For example, almost identical to the knockout mice (*Rbfox1*^-^/^-^) with central nervous system (CNS)-specific deletion of *Rbfox1*, mice with heterozygous deletion (*Rbfox1*^+^/^-^) showed a dramatically increased susceptibility to induced seizures [24]. The loss of Rbfox1 was found to cause an increase in excitability of the neuronal population of the dentate gyrus resulting from an altered synaptic function. Martin et al. reported a 160 kb cryptic deletion of *RBFOX1* within the breakpoint involved in a translocation between the short arms of chromosomes 15 and 16 in a female with autism, epilepsy, and GDD [10]. Accompanied with this heterozygous deletion, reduced mRNA expression in the individual’s lymphocytes was showed, demonstrating the functional consequence of the *RBFOX1* deletion in multiple tissues. In a mouse model of Facioscapulohumeral muscular dystrophy (FSHD), Pistoni et al. recently showed that reducing Rbfox1 expression inhibits muscle differentiation [25].

There are several weaknesses in this study. 1. Ascertainment bias exists in our cohort since many of the patients were referred for CMA analysis because of unexplained neurodevelopmental and neuropsychiatric presentations. 2. Intragenic deletions of *RBFOX1* were observed in some individuals with apparently normal phenotypes although incomplete penetrance of *RBFOX1* defects exist [20,21] (http://projects.tcag.ca/variation/). 3. Most of the intragenic deletions of *RBFOX1* in our patients affected only introns or the noncoding exons although point mutations or intragenic deletions of *RBFOX1* were considered pathogenic in majority of the previous studies [7,9-11,14-19]. 4. The inheritance pattern(s) of the intragenic deletions of *RBFOX1* in this study could not be determined in majority of them due to unavailability of parental samples and Patient 5 who inherited the deletion from her mother, the only determined inheritance of the deletion in this study, showed a different phenotype compared with her mother [15] although variable expressivity might exist.

In conclusion, the current study strongly supports the associations of intragenic deletions of *RBFOX1* with a diversity of neurodevelopmental and neuropsychiatric disorders, and possibly other clinical features. Our data expend the spectrums of both the genomic abnormalities of *RBFOX1* and phenotypic presentations associated with mutated *RBFOX*.

## Material and methods

The DNA samples used for the current study came from 2,124 consecutive pediatric patients referred for chromosomal microarray analysis (CMA) in our laboratory. Each child had one or more of the following clinical findings at the time of referral: intellectual disability, developmental delay, multiple congenital anomalies (MCA), autism spectrum disorders (ASD), dysmorphism, seizures, learning disabilities or speech delays. Informed consents of all patients analyzed in this study were obtained from the parents and the institutional review board of KingMed Genome Diagnostic Laboratory approved this study protocol. The CMA platform used in this study is the Agilent Human Genome Microarray Kit 244K which contains about 244,000 probes across human genome including 186 probes encompassing *RBFOX1* (Agilent Technologies, Santa Clara, CA). All CMA tests were performed and analyzed following protocols described previously [26]. The genomic coordinates of these CNVs in this study were based on the human genome annotation of hg18 build. For confirmation of the abnormal CMA results, quantitative real time PCR (qPCR) methods were performed using three different sets of primers targeting to the deleted regions of *RBFOX1* as described previously [27]. The qPCR method was also applied to test the 5 available parental samples (2 couples and 1 single parent) for determination of the inheritance pattern of the genomic abnormalities in their respective children (Table 1). In addition, all the 14 individuals were previously tested by standard chromosome analysis (G-banding) with negative results and some of them were evaluated by specific genetic tests.

## Competing interests

The author declares that he has no competing interests.
